# Cell-free regenerative medicine: identifying the best source of mesenchymal stem cells for skin therapy in Systemic Sclerosis

**DOI:** 10.3389/fcell.2025.1518412

**Published:** 2025-02-19

**Authors:** Filomena Napolitano, Valentina Giudice, Vittoria D’Esposito, Nella Prevete, Pasqualina Scala, Amato de Paulis, Carmine Selleri, Pietro Formisano, Francesca Wanda Rossi, Nunzia Montuori

**Affiliations:** ^1^ Department of Translational Medical Sciences, University of Naples Federico II, Naples, Italy; ^2^ Department of Medicine, Surgery and Dentistry “Scuola Medica Salernitana”, University of Salerno, Salerno, Italy; ^3^ Hematology and Transplant Center, University Hospital “San Giovanni di Dio e Ruggi d'Aragona”, Salerno, Italy; ^4^ Institute for Experimental Endocrinology and Oncology “G. Salvatore” – National Research Council (IEOS – CNR), Naples, Italy; ^5^ Center for Basic and Clinical Immunology Research (CISI), WAO Center of Excellence, University of Naples Federico II, Naples, Italy

**Keywords:** mesenchymal stem cells, secretome, adipose tissue, bone marrow, Wharton’s jelly, umbilical cord, SSc fibroblasts

## Abstract

**Introduction:**

Systemic Sclerosis (SSc) is a rare chronic systemic autoimmune disease characterized by fibrosis of the skin and internal organs and vasculopathy. Raynaud’s phenomenon is typically the earliest clinical manifestation accompanied by skin inflammation, finger ulcers, and organ manifestations, including pulmonary fibrosis. There is an urgent need for the development of effective targeted therapeutic intervention for SSc patients. A greater focus has been placed on bioactive factors secreted by Mesenchymal Stem Cells (MSCs), with immunomodulatory and regenerative potentials. Current data report a different secretion profile of MSCs, depending on the tissue of origin. Understanding of the secretion profile of different MSCs is necessary to identify the most efficient and useful source for SSc treatment.

**Methods:**

We analyzed the content of MSC-conditioned media (MSC-CM) obtained from MSCs isolated from adipose tissue (AT), bone marrow (BM), Wharton’s jelly (WJ), and cord blood (CB) by ELISA method, and their effects on the wound healing process by fibroblast proliferation, migration, and ECM deposition assays, to compare regenerative potential of different MSC populations.

**Results:**

WJ-MSC-conditioned medium (CM) and BM-MSC-CM show a greater regenerative profile, compared to CB-MSC-CM and AT-MSC-CM, due to the abundance of growth factors and immunomodulatory cytokines and the effects on fibroblast functions. In SSc fibroblasts, WJ-MSC-CM significantly promotes fibroblast-mediated wound healing processes and VEGF expression, compared to BM-MSC-CM.

**Discussion:**

Our data indicate that WJ-MSC-CM could be considered an appealing strategy to both topical and systemic administrations in SSc patients.

## Introduction

In the last decade, Mesenchymal Stem Cells (MSCs) have attracted considerable attention from scientific researchers, as promising candidates in the field of regenerative medicine (Margiana et al., 2022). MSCs are adult multipotential progenitors that can be isolated from several tissues, including adipose tissue, bone marrow (BM), embryonic tissues, dental pulp, tendon, and muscle (Naji et al., 2019). Following pioneering studies performed by Friedenstein et al., who has isolated adherent fibroblast-like clonogenic cells from the BM, the International Society for Cellular Therapy (ISCT) has established minimal criteria to define MSCs: (i) cells must be plastic adherent in standard culture conditions; (ii) must express CD105, CD73 and CD90, and lack expression of CD45, CD34, CD14 or CD11b, CD79α or CD19 and HLA-DR surface molecules; and (iii) must differentiate *in vitro* towards osteoblasts, adipocytes and chondroblasts (Friedenstein et al., 1970; Dominici et al., 2006).

MSCs can represent a potential effective therapy for autoimmune-related fibrotic skin diseases, especially for Systemic Sclerosis (SSc), where MSCs could modulate immune responses, suppress inflammatory processes, and inhibit over-activated fibrotic pathways (Zhang et al., 2023). Preclinical SSc models have provided significant insight into the use of MSCs in rheumatological diseases (Loisel et al., 2023). Notably, intravenous MSC injection improves skin fibrosis and vasculopathy, and reduces ulceration and pain in ongoing clinical trials (Farge et al., 2022; Daumas et al., 2022). Most trials employ BM-derived MSCs, while only a few of them are from adipose tissue, despite these cells can be obtained in a less invasive and painful way and can longer retain their stem cell phenotype (Zhu et al., 2008). Local infusion of MSCs from adipose tissue with hyaluronic acid solution reduces skin fibrosis and improves mouth opening in SSc patients (Scuderi et al., 2013; Onesti et al., 2016), whereas intravenous administration of umbilical cord-derived-MSCs, in combination with plasma exchange and cretinumab, can improve lung function in SSc patients (Zhang et al., 2017; Alip et al., 2024). Fat grafting, rich in MSCs, improved microstomia and microcheilia derived from SSc (Blezien et al., 2017). The MANUS (Mesenchymal stromal cells for Angiogenesis and Neovascularization in digital Ulcers of Systemic Sclerosis) Trial enrolled patients with SSc with refractory digital ulcers and demonstrated that intramuscular injections of bone marrow-derived MSCs improved hand functions and quality of life (van Rhijn-Brouwer et al., 2018).

Before translation to extensive clinical application of MSC infusion, there are still several challenges related to cell therapies, including tissue of origin, standardization of cell preparation according to good manufacturing practices (GMP), and potential risks of immune rejection and tumorigenicity (Sanz-Nogués and O'Brien, 2021; Musiał-Wysocka et al., 2019; Han et al., 2019). To overcome these issues, MSC-conditioned medium (MSC-CM), also known as secretome, can be used as an innovative approach of cell-free regenerative medicine for SSc. Indeed, MSC efficacy is mostly related to secretion of several factors relevant to immune modulation and tissue repair (Gunawardena et al., 2019). MSC-CM contains a wide range of bioactive molecules/factors, such as growth factors, cytokines, and chemokines, with various anti-inflammatory and immunoregulatory effects. These molecules can regulate adaptive and innate immune cells, wound healing, and tissue damage repair, and certain secreted chemokines can also exert antibacterial and antitumor activities (Vizoso et al., 2017; Chang et al., 2021).

Recent clinical studies highlight the therapeutic potential of MSC-CM over direct use of stem cells. MSC-CM is being studied in a variety of regenerative therapies, such as bone regeneration in maxillary sinus floor elevation and multiple sclerosis using bone marrow-derived MSC-CM, or hair growth using adipose tissue-derived MSC-CM (Katagiri et al., 2017; Legiawati et al., 2023). However, there are many open questions on the use of MSC-CM in clinical practice, such as the choice of the most appropriate source of MSCs, or standardization of experimental procedures and culture conditions.

In this study, we explored the biology of MSC-CM obtained from MSCs derived from adipose tissue (AT-MSCs), bone marrow (BM-MSCs), Wharton’s jelly (WJ-MSCs), and cord blood (CB-MSCs). First, we assessed the maintenance of MSC phenotype and morphology, and we subsequently analyzed secreted trophic factors, cytokines, and chemokines, to compare regenerative potential of different MSC populations. Lastly, we studied the effects of MSC-CM on dermal and gingival fibroblasts, to select the specific MSC source of bioactive factors able to promote tissue repair mechanisms in SSc fibroblasts.

## Materials and methods

### Ethic statement

AT-MSCs were obtained from mammary tissues from three healthy women (aged 20, 34, and 55 years old) undergoing surgical mammary reduction not performed for neoplastic, metabolic, or endocrine diseases, in accordance with protocols approved by the Institutional Review Board of University of Naples “Federico II” (prot. no. 138/16). BM-MSCs were obtained from whole BM blood from three healthy male donors (aged 24, 35, and 52 years old), while WJ-MSCs and CB-MSCs from matched pregnant females (aged 46 and 31 years old) after informed written consent obtained in accordance with protocols approved by Ethic Committee “Campania Sud”, Brusciano, Naples, Italy (prot./SCCE no. 24988). SSc fibroblasts were isolated from the skin of three women (aged 76, 68 and 55 years old) affected by limited cutaneous SSc with lung fibrosis, diagnosed at Department of Translational Medical Sciences, University of Naples “Federico II”, Naples, Italy from January 2020 to December 2023, after written informed consent obtained in accordance with protocols approved by the Institutional Review Board of University of Naples “Federico II” (prot. no. 348/2018, approved on 14 March 2018).

### Human cell cultures

For AT-MSCs isolation, biopsies were fragmented and digested by collagenase solution, as previously described (Ambrosio et al., 2022), then cells were cultured in DMEM-F12 (Lonza, Basel, Switzerland) supplemented with 10% FCS (Gibco, Thermo Fisher Scientific, Waltham, Massachusetts, United States), 1% L-Glutamine (Corning, Corning, New York, United States), and 1% penicillin/streptomycin solution (Corning), and incubated at 37°C in an atmosphere of 5% CO_2_ and 95% relative humidity.

For BM-, WJ-, and CB-MSCs, whole blood was directly seeded at a concentration of 50.000 total nucleated cells/cm^2^ in a T75 flask containing minimum essential medium alpha (α-MEM; Gibco) supplemented with 1% Glutagro™ (Corning), 10% Fetal Calf Serum (FCS) and 1% penicillin/streptomycin, and incubated at 37°C in an atmosphere of 5% CO_2_ and 95% relative humidity, as previously described (Scala et al., 2023a). After 48 h, non-adherent cells were removed, medium replaced, and adherent cells were detached when 70%–80% confluent, using 0.05% trypsin with 0.53 mM EDTA. After washed and counted using Trypan Blue (Sigma Aldrich, Milan, Italy), cells were seeded at a density of 4,000 cells/cm^2^ in a medium containing α-MEM supplemented with 1% Glutagro™ (Corning), 10% FCS (Gibco), and 1% penicillin/streptomycin solution (Corning) for further studies or were stored in FCS (Gibco) with 10% DMSO (Sigma-Aldrich) at −80°C until use. Cells were routinely screened for *mycoplasma* contamination.

Experiments involving AT-MSCs, BM-MSCs, WJ-MSCs, and CB-MSCs were performed using cells in the passage number range from 1 to 4.

BJ (human foreskin fibroblasts; ATCC CRL-2522) and HGF-1 (human gingival fibroblasts; ATCC CRL- 2014) were from ATCC (LGC Standards, Milan, Italy) and were grown in DMEM (Lonza) with 1% L-Glutamine (Corning), 10% FCS (Gibco), and 1% penicillin/streptomycin solution (Corning). Primary fibroblasts isolated from punch biopsies of SSc patients were mechanically dissociated under a light microscope and trypsinizated, as previously described (Rossi et al., 2015). Cells were plated and cultured as monolayers in DMEM (Lonza) supplemented with 10% FCS (Gibco), 1% L-Glutamine, and 1% penicillin/streptomycin solution (Corning), at 37°C in an atmosphere of 5% CO_2_ and 95% relative humidity.

### Immunophenotypic characterization of MSCs

Immunophenotyping of MSCs was performed according to ISCT guidelines and as previously reported (Scala et al., 2023a), using the following antibodies for surface marker staining: 2.5 μL of fluorescein isothiocyanate (FITC) - conjugated anti-CD90 or 10 μL of FITC - conjugated anti-HLA-DR; 5 μL of allophycocyanin (APC) - conjugated anti-CD73; 10 μL of phycoerythrin (PE) - conjugated anti-CD105 or 10 μL of PE - conjugated anti-CD34; and 10 μL of phycoerythrin cyanin 7 (PC7) – conjugated anti-CD45 or 10 μL of PC7 – conjugated anti-CD14 (all antibodies were from Beckman Coulter, Fullerton, CA, United State) (Scala et al., 2023a; Manzo et al., 2022). A minimum of 1 × 10^5^ cells were stained with conjugated antibodies and were incubated at room temperature (RT) for 20 min in the dark. Afterwards, samples were washed with 3 mL of phosphate buffer saline (PBS, Gibco), and resuspended in 300 µL of the same buffer for acquisition. Single-color controls for each fluorochrome used and an unstained sample as negative control were employed for setting PMT voltages and for compensation calculation. Acquisition was carried out using a BD FACSVerse flow cytometer (Becton Dickinson, BD, NJ, United State) equipped with blue (488 nm) and red lasers (628 nm), and BD FACSuite software (BD Biosciences). All samples were run using the same PMT voltages, and a minimum of 30,000 events were recorded. FlowJo software (v.10.7.1, LLC, BD Biosciences) or Kaluza Analysis Flow Cytometry Software v2.1.1 (Beckman Coulter) were used for post-acquisition compensation and analysis. Gating strategies used for flow cytometry data analysis are reported in Supplementary Material.

For MSC characterization, cells were first identified using linear parameters (forward scatter area (FSC-A) vs. side scatter area (SSC-A), and double cells were then excluded (area vs. height, FSC-A vs. FSC-H). On single cells, CD90, CD45, CD105, CD73, HLA-DR, CD14, and CD34 expression was investigated. Expression of each marker on single cells was reported using histograms and using unstained samples as negative controls.

### Conditioned medium preparation and collection

Human MSCs from AT, BM, WJ, and CB were seeded at 5 × 10^5^ cells/p60 plates and incubated at 37°C and 5% CO_2_ for 24 h to allow cell adhesion. Prior to the generation of secretome, growth medium was discarded, and cells were washed three times with PBS to remove serum proteins. Then cells were cultured in basal medium in the absence of serum. After 24 h of incubation, supernatants were collected and centrifugated at 5,000 rpm for 10 min to remove cellular debris. The conditioned media were stored at −80°C until use.

### ELISA for cytokines, chemokines, and growth factors secreted by MSCs

MSC-CM were screened for the concentration of interleukin (IL)-1ra, IL-1b, IL-2, IL-4, IL-6, IL-8, IL-9, IL-10, IL-13, IL-17A, basic fibroblast growth factor (FGF), eotaxin, granulocyte-colony stimulating factor (G-CSF), granulocyte macrophage-colony stimulating factor (GM-CSF), interferon-γ (IFN-γ), interferon-γ inducible protein 10 (IP-10), monocyte chemoattractant protein-1 (MCP-1), macrophage inflammatory protein-1 (MIP-1)α, MIP-1β, C–C motif chemokine ligand 5 (CCL5)/RANTES, TNF-α, platelet-derived growth factor (PDGF-BB) and vascular endothelial growth factor (VEGF) using the Bio-Plex Pro Human Cytokine Grp I Panel 27-Plex kit (cat. no. M500KCAF0Y) according to the supplier’s instructions. The magnetic bead-based assay was performed on a Bio-Plex 200 analyzer with Bio-Rad Bio-Plex Manager (Bio-Rad, Hercules, CA, United States).

ELISA kits (from Elabscience, Wuhan, China) were used to determine protein levels of SDF-1, EGF, HGF, and TGF-β1.

### Cell proliferation assay

BJ and HGF-1 cells were serum-starved overnight using DMEM 0.1% BSA, plated at 5 × 10^3^ cells/well in 96-well plates, and then treated with MSC-CM. Cells treated with basal medium were used as a negative control. Cell proliferation was measured at different time points, 0, 24, 48, 72, and 144 h. At the end of incubation, 20 μL/well of CellTiter 96^®^ AQueous One Solution Cell Proliferation Assay (Promega Corporation, Madison, WI, United State), a colorimetric method for determining the number of proliferating viable cells, was added. After incubation at 37°C for 2 h, absorbance was determined by an ELISA reader (Bio-Rad) at 490 nm wavelength according to manufacturer’s instructions.

### Cell cycle analysis

For cell cycle analysis, BJ and HGF-1 cells were plated in 6-well plates (Corning) at a density of 3 × 10^4^ cells per well, serum-starved for 24 h, and treated with medium alone (negative control), AT-MSC-CM, BM-MSC-CM, and WJ-MSC-CM. After 24 h of incubation at 37°C and 5% CO_2_, cells were permeabilized with ice-cold 70% ethanol in phosphate-buffer saline, stored at −20°C overnight, resuspended in phosphate-buffer saline containing 50 μg/mL propidium iodide and 100 mg/mL RNase, incubated at 30°C for 30 min, and analyzed with a FACS Calibur cytofluorimeter using CellQuest software (BD Biosciences, Mississauga, ON, Canada).

### β-Galactosidase (β-gal) staining

Cellular senescence was evaluated after the treatment with medium alone, AT-MSC-CM, BM-MSC-CM, and WJ-MSC-CM for 72 h at 37°C and 5% CO_2_, both in BJ and HGF-1 cells. Cells were washed in PBS, fixed for 5 min at room temperature in 3% formaldehyde, washed twice in PBS, and incubated overnight at 37°C in an atmosphere of 5% CO_2_ and 95% relative humidity with fresh senescence-associated (3-Gal (SA-,3-Gal) stain solution. This solution is composed of 1 mg of 5-bromo4-chloro-3-indolyl P3-D-galactoside (X-Gal) per ml; 40 mM citric acid; sodium phosphate, pH 6.0; 5 mM potassium ferrocyanide; 5 mM potassium ferricyanide; 150 mM NaCl; 2 mM MgCl_2_. The microscopic images were captured using an inverted microscope (Leica DMi1) equipped with camera (FLEXACAM C1, Leica). The number of SA-β-gal positive cells was determined by counting three random fields for each sample and expressed as a percentage of all counted cells.

### Cell migration assay

Cell migration was evaluated using a modified Boyden chamber technique (Rossi et al., 2015). Briefly, 25 µL of medium supplemented with 1% FCS, 2.5% FCS, 5% FCS, and 10% FCS were placed in triplicate in the lower compartment of a microchemotaxis chamber (NeuroProbe, Cabin John, MD, United State), and then were covered with 8-µm-pore polycarbonate membranes coated with 10 μg/mL of fibronectin (Corning Inc., Corning, NY, United State). 50 μL of cell suspension (5 × 10^3^/well) treated or not with MSC-CM for 24 h at 37 °C and 5% CO_2_, were resuspended in basal medium and loaded into the upper compartments. The chemotactic chamber was then incubated for 24 h at 37°C in a humidified incubator with 5% CO_2_. Then, the membrane was removed, the upper side was washed with PBS, and cells attached to the lower filter surface were fixed, stained with May-Grünwald-Giemsa, mounted on a microscope slide with Cytoseal (Stephens Scientific, Springfield, NJ), and counted. In each experiment, 10 fields/triplicate filters were measured at ×40 magnifications.

### ECM deposition

ECM deposition was evaluated by ELISA protocol developed by our group (Scala et al., 2023b). BJ and HGF-1 cells were plated in 96-well plates (Corning) at a density of 5 × 10^3^ cells per well and treated with medium alone (negative control), TGF-β (positive control), and MSC-CM. After 24 h of incubation at 37°C and 5% CO_2_, proteins were fixed by acetone/methanol (v/v), for 10 min at 22°C, incubated in 0.5% PBS/BSA and 0.2% Tween 20 for 30 min at 22°C to minimize aspecific binding sites, and washed in PBS. Goat polyclonal anti-vitronectin (2 μg/mL) antibody, and mouse monoclonal anti-fibronectin (2 μg/mL) and anti-collagen type 1 (2 μg/mL) antibodies were added for 1 h at 22°C. After three washes in PBS, plates were incubated with HRP secondary antibodies for 30 min at 22°C. After three washes in PBS, the substrate was added (1 mg/mL OFD, 0.1 mol/L citrate buffer [pH 5], and 0.006% H_2_O_2_), and plates were incubated for 30 min at 37°C in the dark. The reaction was then stopped by 1 M sulfuric acid (H_2_SO_4_), and absorbance was read at 450 nm by an ELISA reader (Bio-Rad).

### Real-time PCR

Quantitative real-time RT-PCR for VEGF isoforms was performed in SSc fibroblasts pre-treated or not with BM-MSC-CM and WJ-MSC-CM for 24 h at 37°C and 5% CO_2._ Total RNA was isolated and retrotranscribed according to the manufacturer’s instructions (Qiagen, GmbH, Hilden, Germany). The RNA samples were stored at −80°C until further analysis. Real-time quantitative PCR was performed on iCycler (Bio-Rad, Hercules, CA, United State) using the PE SYBR Green PCR kit (Applied Biosystems, Foster City, CA, United State). Normalization was performed using β-actin mRNA levels. VEGF-A primer sequences were forward 5′-GTG​AAT​GCA​GAC​CAA​AGA​AAG-3′ and reverse 5′-AAA​CCC​TGA​GGG​AGG​CTC-3′; VEGF-B primer sequences were forward 5′- TGT​CCC​TGG​AAG​AAC​ACA​GCC-3′ and reverse GCC​ATG​TGT​CAC​CTT​CGC-3′; VEGF-C primer sequences were forward 5′-ATG​TTT​TCC​TCG​GAT​GCT​GGA-3′ and reverse 5′-CAT​TGG​CTG​GGG​AAG​AGT​TT-3′; VEGF-D primer sequences were forward 5′-GTA​TGG​ACT​CTC​GCT​CAG​CAT-3′ and reverse 5′-AGG​CTC​TCT​TCA​TTG​CAA​CAG-3′.

### Scratch assay

SSc fibroblasts were seeded into a 6-well culture plate (Corning) using DMEM containing 10% FCS and incubated for 12 h at 37°C and 5% CO_2_ to create a confluent monolayer. Cells were then scraped with a p200 pipette tip in a straight line to create a “scratch”. Debris was removed, and the edge of the scratch was smoothed by washing cells once with 2 mL growth medium. Cells were then incubated at 37°C and 5% CO_2_ with basal medium (as a negative control) or MSC-CM for different time points, at baseline (T = 0) and 24 h. The microscopic images were captured using under inverted microscope (Leica DMi1) equipped with camera (FLEXACAM C1, Leica) and gap measurement at each time point were measured using ImageJ software (National Institutes of Health, Bethesda, Maryland, United State). Three measurements of length for each digital picture were performed to make an accurate measurement on the image.

### ELISA for measurement of VEGF-A

VEGF-A Human ELISA Kit (Elabscience, Catalog No E-EL-HO111, Wuhan, China) is an *in vitro* enzyme-linked immunosorbent assay, which was performed for the quantitative measurement of human VEGF-A in cell lysates and conditioned media. According to the manufacturer’s protocol, cell lysates and conditioned media from cells treated with or without BM-MSC-CM and WJ-MSC-CM for 24 h at 37°C and 5% CO_2_ were collected. The samples were added to the micro-ELISA plate wells and VEGF present in a sample was bound to the wells by the immobilized antibody. The wells were washed and biotinylated anti-Human VEGF antibody was added. After washing away unbound biotinylated antibody, HRP-conjugated streptavidin was pipetted to the wells. The wells were washed and a TMB substrate solution was added. The enzyme-substrate reaction was terminated by the addition of Stop Solution. The optical density was measured at 450 nm by an ELISA reader (Bio-Rad) and the concentration of VEGF-A in the samples were calculated by comparing optical density of the samples to the standard curve.

### Masson’s trichrome staining

Fibroblasts isolated from 3 SSc patients were suspended in growth media and seeded onto microscope slides (5 × 10^3^/slide) placed in 24 well culture plates. After obtaining the required confluency, cells were treated with AT-MSC-CM, BM-MSC-CM, and WJ-MSC-CM for 24 h at 37°C and 5% CO_2_ and scraped with a p20 pipette tip in a straight line to create a scratch. On attaining 24 h of incubation, the media were discarded, and the cells were washed 3 times in PBS 1X at room temperature. Masson’s Trichrome staining in cultured fibroblasts was adapted to Masson’s Trichrome Stain Kit, Artisan (Agilent Dako, Santa Clara, CA, United State) used to identify muscle, collagen fibers, fibrin and erythrocytes in tissue sections. Cells were fixed in Bouin’s solution containing 0.9% Picric acid, 23.8% formaldehyde, and 4.8% acetic acid in deionized water which has been preheated to 58°C for 15 min. After wash of slides with distilled water, Weigert’s Hematoxilin A solution containing 1% hematoxilin and 95% alcohol was added for 5 min. After 3 washes in distilled water, Weigert’s Hematoxilin B solution containing 1.16% Ferric chloride and 1% hydrochloric acid in deionized water was added for 1 min. After 3 changes of distilled water, Biebrich Scarlet Acid Fuchsin (0.1% Acid fuchsin, 0.9% Biebrich Scarlet and 1% acetic acid in deionized water containing 1% Triton X-100) was added for 5 min. After 3 changes of distilled water, Phosphotungstic Phosphomolybdic Acid for 2 min and Aniline Blue for 5 min were added. After 2 changes of distilled water, the images were captured under inverted microscope (Leica DMi1) equipped with camera (FLEXACAM C1, Leica).

### Statistical analysis

Each value represents the mean ± SEM of replicate from different samples isolated from human specimens, as described in figure legend. Indicated *p* values were obtained using the Student’s t-test. Differences were considered statistically significant when *p < 0.05; **p < 0.01; ***p < 0.001. Parametric analysis of variance, by one-way ANOVA, was used to analyze experimental data obtained from multiplex and ELISA assays. All statistical analyses were performed using GraphPad Prism 7.0 software (GraphPad Software Inc., La Jolla, CA, United State).

## Results

### MSC characterization

**FIGURE 1:**
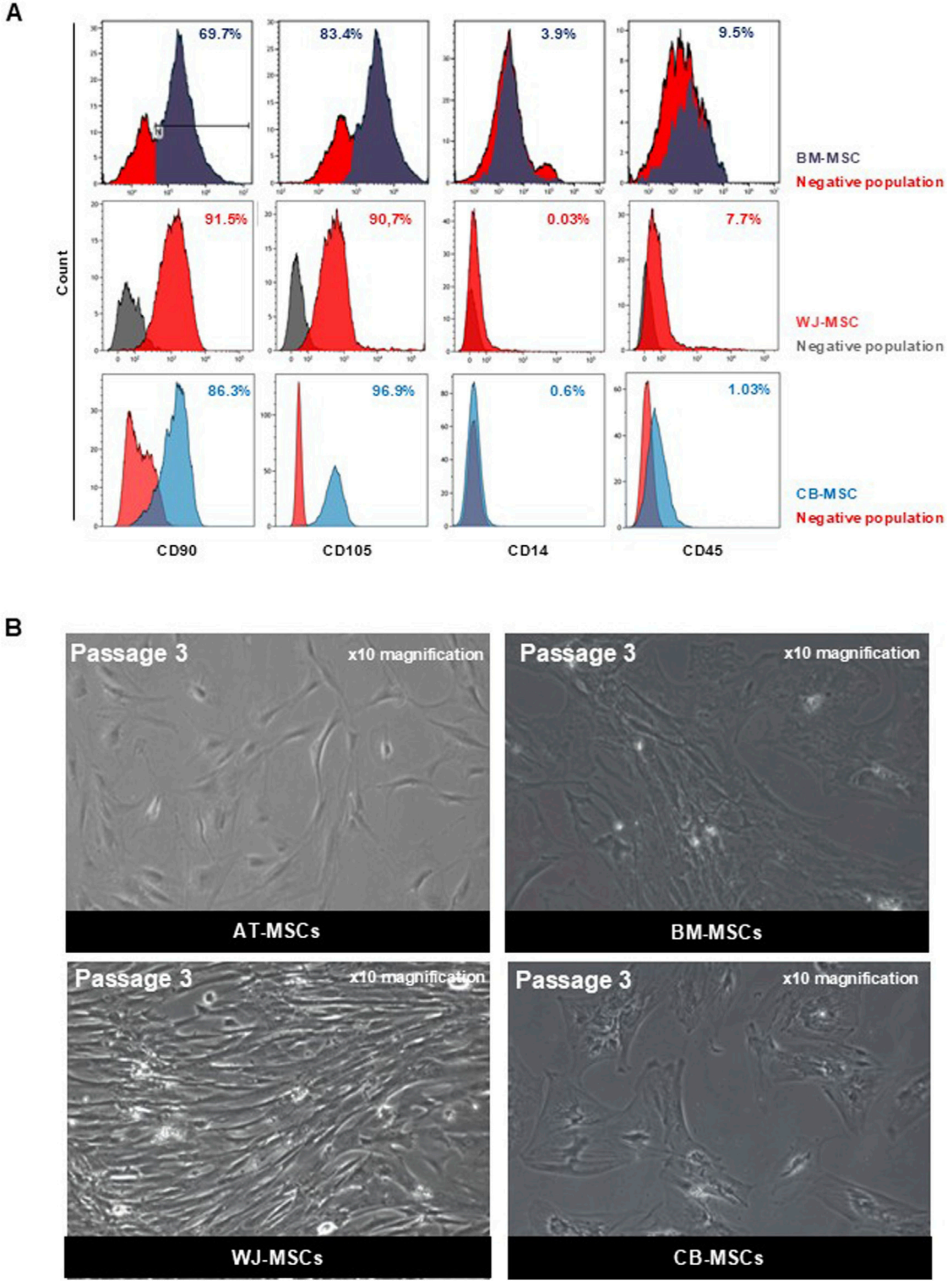
Characterization of MSCs. **(A)** Representative dot plot from FACS analysis of BM-MSCs, WJ-MSCs, and CB-MSCs stained for FITC-anti-CD90, PE-anti-CD105, APC-Cy7-anti-CD14, and APC-Cy7-anti-CD45. **(B)** Representative microscopic image (magnification ×10) of AT-MSCs, BM-MSCs, WJ-MSCs, and CB-MSCs.

Cells used in these experiments have been validated for their mesenchymal phenotype according to the ISCT criteria. AT-MSCs have previously differentiated towards adipogenic and osteogenic lineage, whereas BM-MSCs, WJ-MSCs, and CB-MSCs have been previously employed for tenogenic, chondrogenic, and myogenic commitment differentiation experiments (Ambrosio et al., 2022; Scala et al., 2023a; Manzo et al., 2022; Scala et al., 2023b; Scala et al., 2022; Lamparelli et al., 2022; Ciardulli et al., 2021; Lamparelli et al., 2021; Ciardulli et al., 2020a; Ciardulli et al., 2020b; Marino et al., 2023). To assess mesenchymal phenotype of cultured MSCs obtained from different sources, flow cytometry immunophenotyping and cell morphology analysis were performed. Flow cytometry analysis of AT-MSCs used in this study was previously performed, confirming that these cells were positive for mesenchymal progenitor cell surface antigens CD90 and CD73 and negative for hematopoietic marker CD45 (Ambrosio et al., 2022). As per ISCT guidelines, BM-MSCs, WJ-MSCs, and CB-MSCs were positive for mesenchymal stem cell markers, such as CD90, CD73 and CD105, while they did not express hematopoietic markers, like CD34, CD14 and CD45, confirming their mesenchymal phenotype retaining after first expansion and culture (Figure 1A).

All these MSCs were plastic adherent in standard culture conditions and had a fibroblast-like spindle shape morphology. However, different morphological shapes were observed from the first passage of cultivation between tissue-specific MSCs, although no heterogeneity was found within cells belonging to the same tissue of origin. In Figure 1B, representative images of each tissue-specific MSCs at the third passage in culture are displayed. AT-MSCs showed small triangular cell shape; BM- and CB-MSCs were large, flattened cells with prominent nucleus, while WJ-MSCs were elongated, fibroblast-like, spindle-shaped cells. Therefore, MSCs from different sources are phenotypically similar, while their morphological heterogeneity underlies their anatomical tissue and embryonic origins, confirming that MSCs are tissue-committed progenitors (Liu et al., 2019).

### BM-MSC- and WJ-MSC-CM could mediate immunoregulatory functions

Secretome of MSCs could be used for treatment of several diseases associated with inflammation, wound healing, infection, and degeneration (Katagiri et al., 2017; Legiawati et al., 2023; Xia et al., 2018; Pouya et al., 2018; Heidari et al., 2018); however, the best cell source for clinical use of secretome is still under debate and published literature is very heterogeneous, as few comparative analyses have been reported (Berebichez-Fridman and Montero-Olvera, 2018). Because variable morphology and viability of studied MSCs were observed by microscopy, we hypothesized that this morphological heterogeneity could have also reflect a secretome tissue-specific variability, thus a different ability to influence and promote tissue repair and immune modulation. Therefore, based on these observations, a secretome analysis by multiplex and ELISA assays was performed.

**FIGURE 2:**
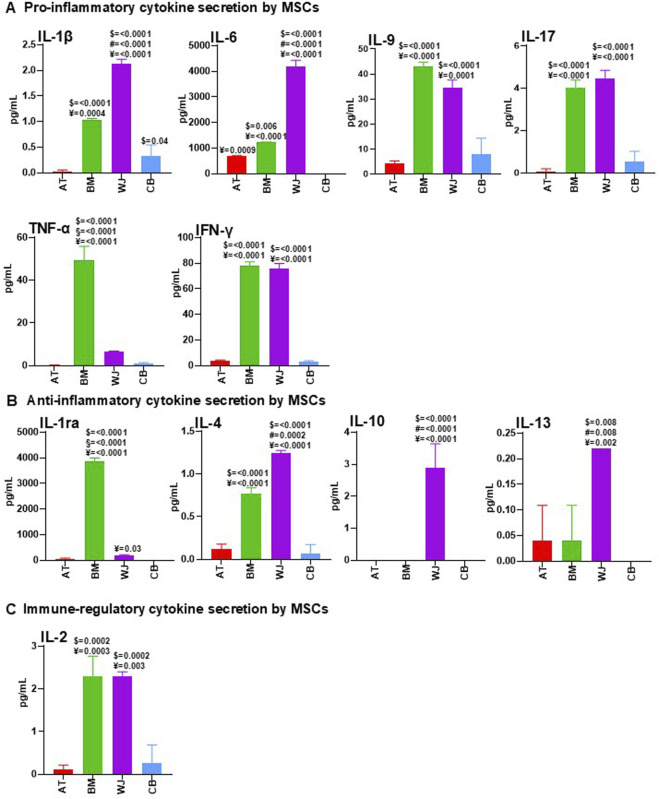
Comparative analysis of cytokine content between AT-MSC-CM, BM-MSC-CM, WJ-MSC-CM, and CB-MSC-CM. AT-MSCs, BM-MSCs, WJ-MSCs, and CB-MSCs were cultured in basal medium. After 24 h, CM from these cells were collected and analyzed for pro-inflammatory **(A)**, anti-inflammatory **(B)**, and immune-regulatory **(C)** cytokine content by using multiplex human cytokine approach or ELISA kits (see Methods). Data were reported as mean of three separate samples. For multiple comparisons, one-way ANOVA was assessed. $ denotes statistically significant values compared with AT-MSC-CM; # denotes statistically significant values compared with BM-MSC-CM; § denotes statistically significant values compared with WJ-MSC-CM; ¥ denotes statistically significant values compared with CB-MSC-CM.

First, to identify cytokines secreted by AT-MSCs, BM-MSCs, WJ-MSCs, and CB-MSCs, supernatants from these cell cultures were analyzed, showing a different pro-inflammatory cytokine secretion profiling between tissue-specific MSCs (Figure 2A). BM-MSC-CM and WJ-MSC-CM contained higher concentrations of IL-1β, IL-9, IL-17, and IFN-γ, compared to AT-MSC-CM and CB-MSC-CM. IFN-γ can act as an anti-fibrotic factor by reducing ECM synthesis and disrupting TGF-β signaling (Cheng et al., 2008), while IL-6 has a wide range of biological activities, including wound healing and angiogenesis (Pu et al., 2019). IL-6 was not detectable in CB-MSC-CM, conversely WJ-MSC-CM contained significantly higher levels of this cytokine compared to AT-MSC-CM and BM-MSC-CM. TNF-α is a major regulator of inflammatory responses, promotes the recruitment of inflammatory and stromal cells, stimulates angiogenesis, and is essential for bone fracture repair (Karnes et al., 2015), and was found at high levels in BM-MSC-CM, as well as IL-1ra. In contrast, WJ-MSC-CM showed an anti-inflammatory profiling with high levels of IL-4, IL-10 -the principal anti-inflammatory human cytokine (Saraiva et al., 2020), and IL-13, as reported in Figure 2B. IL-2 plays a central role in regulatory T cell (Treg) development and homeostasis (Harris et al., 2023), and elevated levels were found in BM-MSC-CM and WJ-MSC-CM (Figure 2C).

Therefore, AT-MSC-CM and CB-MSC-CM were a dispensable source of cytokines, whereas BM-MSC-CM and WJ-MSC-CM contained high levels of immunoregulatory molecules, with WJ-MSC-CM presenting a perfect balance between anti-inflammatory and pro-inflammatory factors, thus creating a cytokine microenvironment that could be crucial for successful healing (Suzdaltseva et al., 2022).

### BM-MSC- and WJ-MSC-CM as a source of proangiogenic chemokines

**FIGURE 3:**
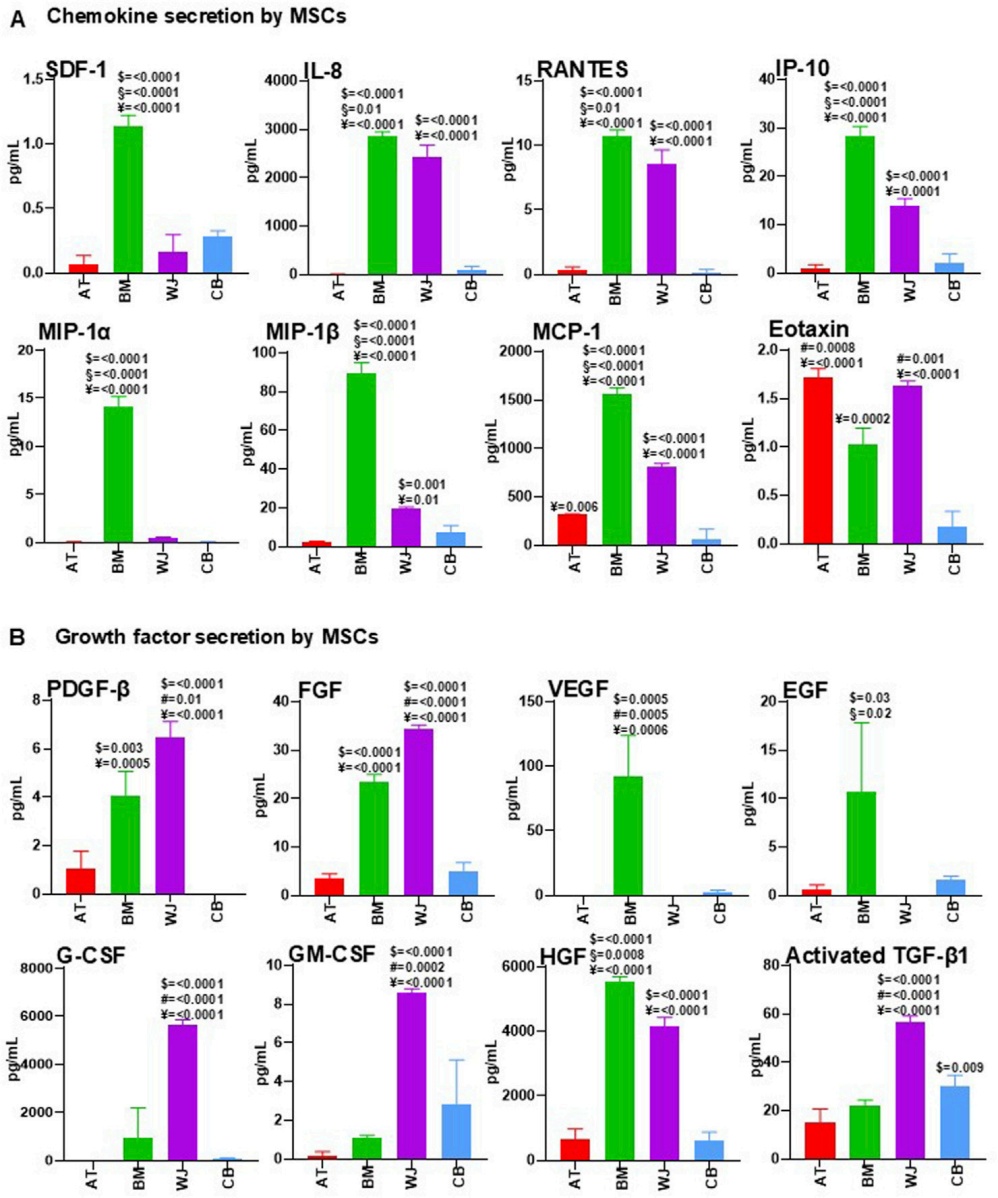
Comparative analysis of cytokine content in AT-MSC-CM, BM-MSC-CM, WJ-MSC-CM, and CB-MSC-CM. AT-MSCs, BM-MSCs, WJ-MSCs, and CB-MSCs were cultured in basal medium. After 24 h, CM from these cells were collected and analyzed for chemokine **(A)** and growth factor **(B)** content by using multiplex human cytokine approach or ELISA kits (see Methods). Values are mean ± SEM of three independent samples. For multiple comparisons, one-way ANOVA was assessed. $ denotes statistically significant values compared with AT-MSC-CM; # denotes statistically significant values compared with BM-MSC-CM; § denotes statistically significant values compared with WJ-MSC-CM; ¥ denotes statistically significant values compared with CB-MSC-CM.

As chemokine profiling can be used to predict *in vivo* therapeutic potential of MSCs (Cuesta-Gomez et al., 2021), chemokines secreted by AT-MSCs, BM-MSCs, WJ-MSCs, and CB-MSCs were measured under pre- and post-conditioning. As reported in Figure 3A, MSCs isolated from different tissues had differential chemokine profiles, as stromal cell-derived factor 1 (SDF-1), also known as CXCL12, was detectable in all investigated CM, with the highest levels in BM-MSC-CM. As SDF-1 secreted by MSCs is crucial for cell migration and angiogenesis (Cun et al., 2021), BM-MSC-CM could represent the most appropriate source of factors for vascularization and wound healing.

IL-8 has evolved from a neutrophil chemoattractant in acute inflammation to a chemotactic for endothelial cells with a major role in angiogenesis (Matsushima et al., 2022). In our study, BM-MSC-CM and WJ-MSC-CM were the main source of IL-8, thus confirming their potential role in vascularization processes. RANTES, also known as CCL5, and alpha chemokine Interferon gamma-induced protein 10 (IP-10) were found in large amounts in BM-MSC-CM and WJ-MSC-CM, while high concentrations of MIP-1α/CCL3 in BM-MSC-CM and of MIP-1β/CCL4 in BM-MSC-CM and WJ-MSC-CM were observed. Monocyte chemoattractant protein-1 (MCP-1), also known as CCL2, were present at high concentration in BM-MSC-CM and WJ-MSC-CM, and a significant concentration was also found in AT-MSC-CM. Eotaxin-1 (CCL11), involved in selective eosinophil recruitment in inflammatory sites (Ivanovska et al., 2020), was present at higher levels in AT-MSC-CM and WJ-MSC-CM compared to BM-MSC-CM and CB-MSC-CM.

Based on these results, BM-MSC-CM and WJ-MSC-CM contained larger amounts of chemokines compared to AT-MSC-CM and CB-MSC-CM, especially those with angiogenic properties (SDF-1, IL-8, MCP-1), that could exert beneficial effects in treatment of vascular diseases, including SSc. However, effects of pro-inflammatory IL-8, RANTES, and Eotaxin-1 should be further investigated.

### BM-MSC- and WJ-MSC-CM are major sources of growth factors

MSC-derived secretome is increasingly proposed as an alternative approach in regenerative medicine, and multiple growth factors have been studied and identified in the secretion profile of MSCs (Ahangar et al., 2020). However, there are still few comparative analyses between trophic factors secreted by tissue-specific MSCs, and in this study, we mainly focused on growth factors involved in tissue repair (Figure 3B). Platelet-derived growth factor (PDGF) activates signals involved in chemotaxis, growth, proliferation, and differentiation, and was significantly expressed by BM-MSC-CM and WJ-MSC-CM, as well as fibroblast growth factor (FGF), with a recognized role in epithelial- and mesenchymal-cell proliferation and angiogenesis. Vascular endothelial growth factor (VEGF) and epidermal growth factor (EGF) were found at high levels in BM-MSC-CM, whereas granulocyte colony-stimulating factor (G-CSF) and granulocyte-macrophage colony-stimulating factor (GM-CSF), two hematopoietic growth factors, were surprisingly most abundant in WJ-MSC-CM compared to other MSC-CM. Hepatocyte growth factor (HGF) is the most frequently used growth factor in MSC therapy, exerting mitogenic, motogenic, anti-apoptotic, morphogenic, and immune regulation functions, and preventing fibrosis while promoting angiogenesis (Meng et al., 2022; Molnarfi et al., 2015; Matsumoto and Nakamura, 2001). In our investigation, HGF was significantly present in BM-MSC-CM and WJ-MSC-CM, confirming the high regenerative potential of these cells. Transforming growth factor-beta (TGF-β) contributes to tissue repair and restoration of normal tissue architecture; however, excessive levels of this factor promote tissue fibrosis leading to organ dysfunction, as occurring in SSc (Varga and Pasche, 2009). Here, TGF-β was higher in fetal MSCs, WJ- and CB-MSCs.

BM-MSC-CM and WJ-MSC-CM are major sources of growth factors, but effects of high TGF-β levels in WJ-MSC-CM should be better investigated.

### Different effects of tissue-specific MSC-CM on fibroblast proliferation and cell cycle progression

To correlate the cytokine profiling and functional effects, we pursued our studies by analyzing the effects of AT-MSC-CM, BM-MSC-CM, and WJ-MSC-CM on human fibroblast cell lines. CB-MSC-CM were excluded from further analyses because previous secretome analysis revealed that, among fetal MSCs, WJ-MSC-CM appeared to be much more effective and promising for treating chronic inflammatory diseases.

**FIGURE 4:**
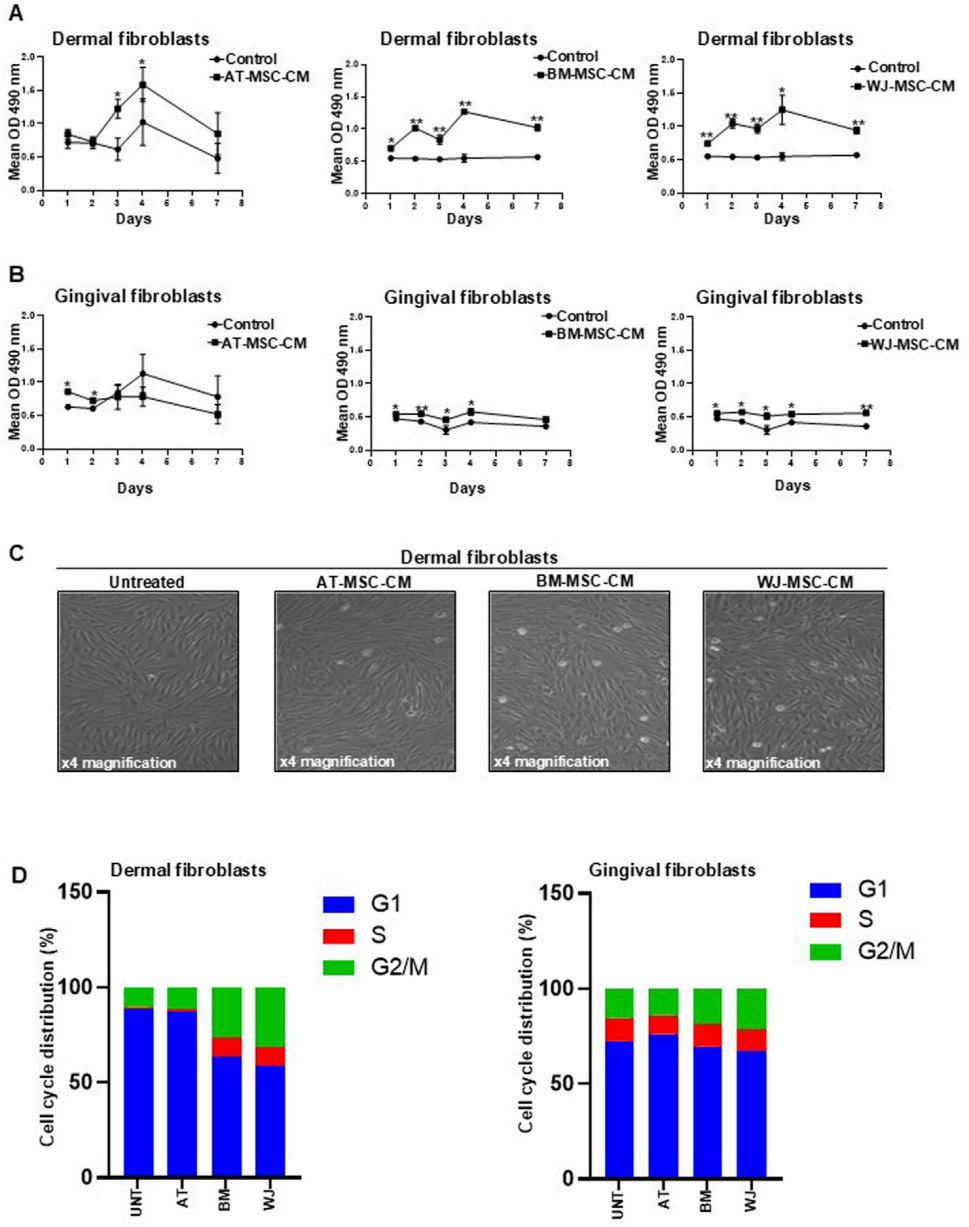
Effects of AT-MSC-CM, BM-MSC-CM, and WJ-MSC-CM on cell proliferation and cell cycle progression in dermal and gingival fibroblasts. **(A)** Dermal fibroblasts and **(B)** gingival fibroblasts were treated with AT-MSC-CM, BM-MSC-CM, and WJ-MSC-CM for 24, 48, 72, and 144 h. Cells treated with medium alone were used as a negative control. Values are mean ± SEM of three experiments. *p < 0.05; **p < 0.001. **(C)** Representative microscopic image of dermal fibroblasts treated for 24 h with AT-MSCs, BM-MSC-CM, and WJ-MSC-CM. Various stages of mitosis are easily distinguished under magnifications ×4. **(D)** Cell cycle distribution of dermal and gingival fibroblasts, treated or not with AT-MSC-CM, BM-MSC-CM, and WJ-MSC-CM, measured by Propidium Iodide (PI) staining by means of flow cytometry at 24 h. The percentage of the cells distributed in G1, S, and G2/M phases was indicated in each panel.

In these experiences, BJ cells, which are fibroblasts established from skin taken from normal foreskin, were used to investigate MSC-CM function in the scenario of skin repair, while human gingival fibroblast HGF-1 were used as cell model of internal organ. Thus, dermal and gingival fibroblasts were grown in culture medium supplemented with 0.1% FCS, as a negative control, and MSC-CM at different time points. In parallel, cells grown in culture medium supplemented with 10% FCS were used as a positive control (data not shown). Representative growth curves of dermal and gingival fibroblasts are shown in Figures 4A, B, respectively. AT-MSC-CM induced significantly cell proliferation in dermal fibroblast proliferation at days 3 and 4 and in gingival fibroblasts at days 1 and 2 compared to the control cells. BM-MSC-CM significantly stimulated cell proliferation at all time points both in dermal fibroblasts and in gingival fibroblasts compared to the control cells. Growth kinetics obtained with BM-MSC-CM was like WJ-MSC-CM which significantly induced cell proliferation at all time points both in dermal fibroblasts and in gingival fibroblast compared to the control cells. These results indicate that BM-MSC-CM and WJ-MSC-CM could exert their beneficial effects on both skin and internal organs, as demonstrated by significant proliferation of dermal and gingival fibroblasts.

The ability of BM-MSC-CM and WJ-MSC-CM to induce dermal fibroblast proliferation in a significant manner compared to AT-MSC-CM is appreciable in Figure 4C by the number of cell divisions (mitosis), evaluated by the morphological characteristics, after only 24 h of treatment. The results agree with secretome composition of these cells as BM-MSC-CM and WJ-MSC-CM was found to be enriched with trophic factors such as cytokines and growth factors, including PDGF, FGF, and HGF.

To better define the mechanisms underlying AT-, BM- and WJ-MSC-CM-mediated fibroblast growth, we measured the distribution of cells in the different phases of the cell cycle by flow cytometry. Both dermal and gingival fibroblasts, treated or not with AT-MSC-CM, BM-MSC-CM, and WJ-MSC-CM for 24 h, were subjected to cell cycle analysis, as shown in Figure 4D. In dermal fibroblasts, AT-MSC-CM exerted inhibitory effects on cell cycle progression, as shown by the reduction in S (1.1%) and G2/M (11.5%) and the accumulation in G1 phase (87.4%), similar to untreated cells that exhibited 10.4% of cells in G2/M and 1.2% of cells in S phases and an accumulation in G1 (88.3%). BM-MSC-CM induced cell cycle progression as demonstrated by cell distribution in S (10%) and G2/M (26.5%) phases against G1 phase (63.4%). In the same way, WJ-MSC-CM stimulated dermal fibroblasts to progress toward S (9.8%) and G2/M (31.4%) phases and reduced cell distribution in G1 phase (58.8%). In gingival fibroblasts, we observed that AT-MSC-CM had no effects on cell cycle progression, as shown by a low percentage of the cells in S (10%) and G2/M (14%) phases and an accumulation in G1 phase (76%), similar to untreated cells that exhibited 15.5% of cells in G2/M and 12.1% of cells in S phases and a prevalent cell cycle distribution in G1 (72.3%). A slight decrease in G1 phase was observed in cells treated with BM-MSC-CM (69.7%) and WJ-MSC-CM (67.1%), as compared to untreated cells. The same cell distribution in S phase (11.5%) occurred after the treatment with BM-MSC-CM and WJ-MSC-CM. Cells in the G2/M population slightly increased after treatment with BM-MSC-CM (18.8%) and WJ-MSC-CM (21.4%), as compared to negative control.

Overall, the data of cell proliferation showed that BM-MSC-CM and WJ-MSC-CM represent mitogenic stimuli in both dermal and gingival fibroblasts, although they preferentially induce cell cycle progression in dermal fibroblasts. It is conceivable that BM-MSC-CM and WJ-MSC-CM could be successfully used for skin application to regenerate and repair tissue damage.

### AT-MSC-CM, BM-MSC-CM, and WJ-MSC-CM protect fibroblasts from cellular senescence

**FIGURE 5:**
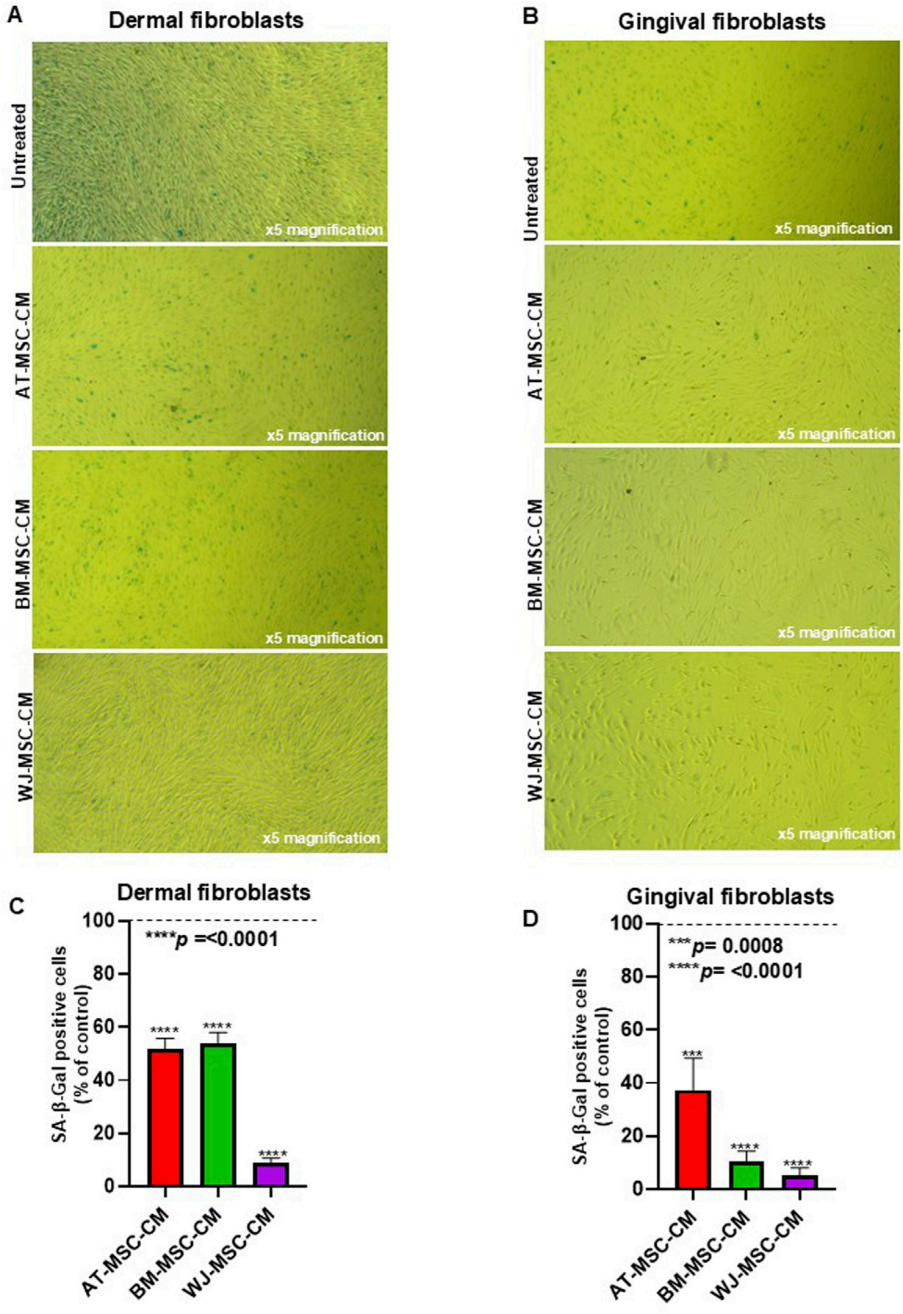
Effects of AT-MSC-CM, BM-MSC-CM, and WJ-MSC-CM on cellular senescence. **(A)** Dermal fibroblasts and **(B)** gingival fibroblasts treated with medium alone (untreated) or AT-MSC-CM, BM-MSC-CM, and WJ-MSC-CM for 72 h at 37°C in a humidified (5% CO_2_) incubator were stained with senescence-associated β-Galactosidase (SA-β-Gal). **(C)** Percentage of SA-β-Gal-positive cells, determined by counting three random fields for each sample, in dermal fibroblasts. **(D)** Percentage of SA-β-Gal-positive cells, determined by counting three random fields for each sample, in gingival fibroblasts.

Cellular senescence is implicated in SSc pathogenesis and mainly involves endothelial cells and fibroblasts. Senescence in these cell types is associated with SSc vasculopathy and fibrosis (Tsou et al., 2022). Since senescence could be responsible for the increased number of pathogenic myofibroblasts in SSc, we aim to analyze the effects of AT-MSCs, BM-MSC-CM, and WJ-MSC-CM on cellular senescence both in dermal and gingival fibroblasts. To this end, cells were treated or not with AT-MSCs, BM-MSC-CM, and WJ-MSC-CM for 72 h and stained with senescence-associated β-Galactosidase (SA-β-Gal), which is widely used marker for *in vitro* senescence study and is linked to the increased lysosome content (Cai et al., 2020). Both BJ and HGF-1 cells varied in terms of the intensity of the SA-β-gal signal after the treatments, as shown in Figure 5A and in Figure 5B. The analysis of percentage of SA-β-Gal-positive cells in dermal fibroblasts, determined by counting the number of blue colored cells, showed that both AT-MSC-CM, BM-MSC-CM, and WJ-MSC-CM significantly reduced cellular senescence as compared to untreated cells. Particularly, AT-MSC-CM and BM-MSC-CM share a similar behavior, whereas WJ-MSC-CM drastically reduced cellular senescence in dermal fibroblasts as compared to control (Figure 5C). In parallel, the analysis of percentage of SA-β-Gal-positive cells in gingival fibroblasts showed a significant reduction of cellular senescence after the treatments, with substantial effects in BM and WJ-MSC-CM-treated cells (Figure 5D).

Ultimately, we demonstrated that MSC-CM-treated cells, particularly BM- and WJ-MSC-CM-treated cells, decreased the accumulation of senescent cells. Furthermore, these data agree with the specific composition of BM-MSC-CM and WJ-MSC-CM and their effects on cell proliferation and cell cycle progression previously described.

### WJ-MSC-CM preferentially induces fibroblast migration

As fibroblast migration is a key step in wound healing process (Cen et al., 2022), we analyzed wound healing potential of secretome from AT-MSC-CM, BM-MSC-CM, and WJ-MSC-CM by migration assay using dermal and gingival cells. These cells were pretreated for 24 h with basal medium, as a control, or MSC-CM, placed inside the chamber, and allowed to migrate towards medium containing increasing concentrations of FCS (1%, 2.5%, 5%, and 10%).

**FIGURE 6:**
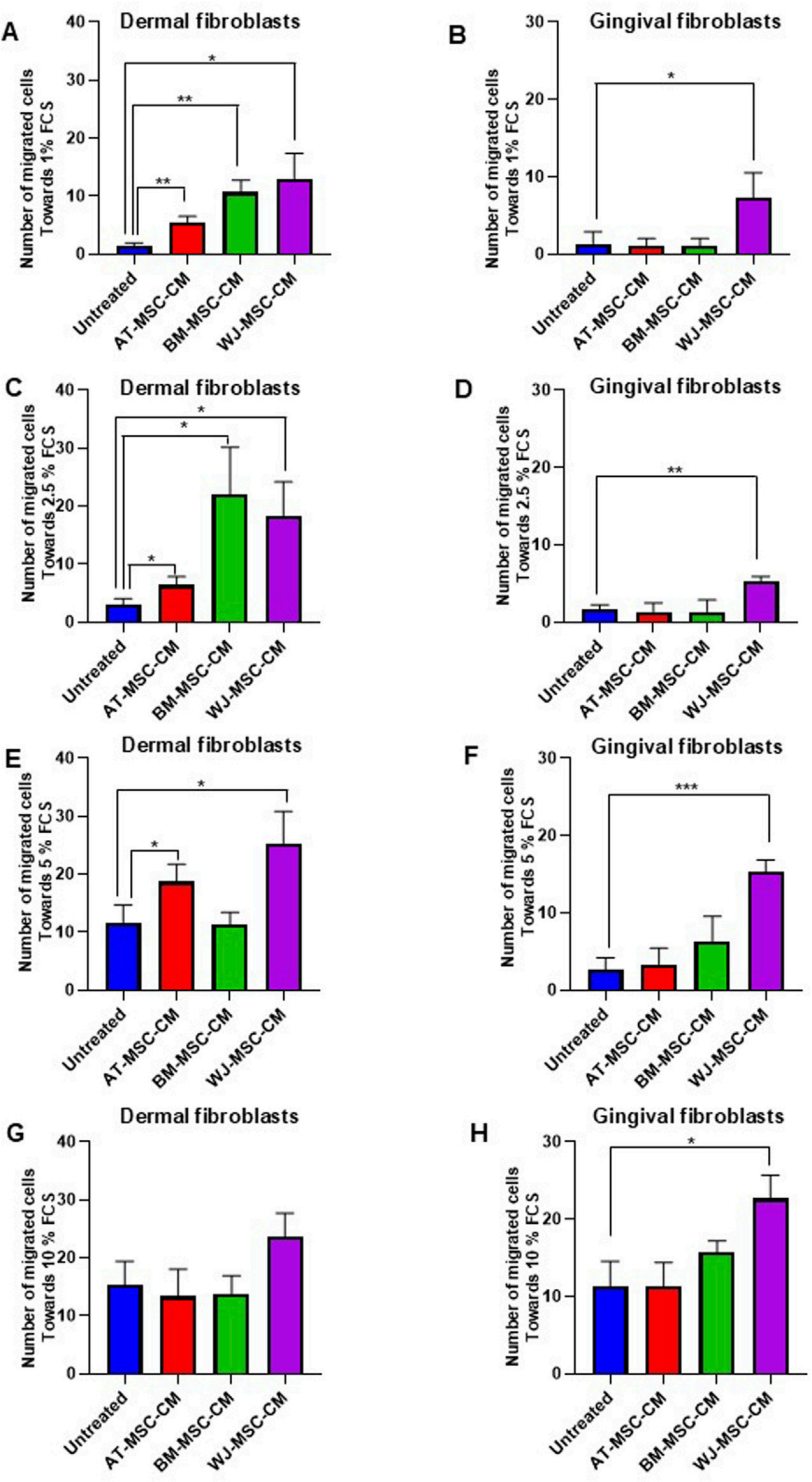
Effects of AT-MSC-CM, BM-MSC-CM, and WJ-MSC-CM on cell migration in dermal and gingival fibroblasts. **(A)** Dermal fibroblasts and **(B)** gingival fibroblasts treated with medium alone and AT-MSC-CM, BM-MSC-CM, and WJ-MSC-CM were allowed to migrate in response to 1% FCS for 24 h at 37°C in a humidified (5% CO_2_) incubator. Error bars represent standard deviation of the mean of triplicate samples within one experiment. *p < 0.05; **p < 0.005. **(C)** Dermal fibroblasts and **(D)** gingival fibroblasts treated with medium alone and AT-MSC-CM, BM-MSC-CM, and WJ-MSC-CM were allowed to migrate in response to 2.5% FCS for 24 h at 37°C in a humidified (5% CO_2_) incubator. Error bars represent standard deviation of the mean of triplicate samples within one experiment. *p < 0.05; **p < 0.005. **(E)** Dermal fibroblasts and **(F)** gingival fibroblasts treated with medium alone and AT-MSC-CM, BM-MSC-CM, and WJ-MSC-CM were allowed to migrate in response to 5% FCS for 24 h at 37°C in a humidified (5% CO_2_) incubator. Error bars represent standard deviation of the mean of triplicate samples within one experiment. *p < 0.05; **p < 0.005; ***p < 0.0005. **(G)** Dermal fibroblasts and **(H)** gingival fibroblasts treated with medium alone and AT-MSC-CM, BM-MSC-CM, and WJ-MSC-CM were allowed to migrate in response to 5% FCS for 24 h at 37°C in a humidified (5% CO_2_) incubator. Error bars represent standard deviation of the mean of triplicate samples within one experiment. *p < 0.05.

As shown in Figure 6A, cell migration towards 1% FCS was significantly stimulated by AT-MSC-CM (*p* = 0.005), BM-MSC-CM (*p* = 0.001), and WJ-MSC-CM (*p* = 0.01) in dermal fibroblasts and the treatment with WJ-MSC-CM resulted in a better performance. In gingival fibroblasts, only WJ-MSC-CM (*p* = 0.04) significantly induced gingival fibroblast proliferation, as compared to untreated cells (Figure 6B). Cell migration towards 2.5% FCS was likewise stimulated by AT-MSC-CM (*p* = 0.03), BM-MSC-CM (*p* = 0.01), and WJ-MSC-CM (*p* = 0.01) in dermal fibroblasts (Figure 6C), whereas only WJ-MSC-CM (*p* = 0.001) enhanced gingival fibroblast proliferation (Figure 6D). The analysis of migrated cell number towards medium concentrations of FCS (5% FCS) showed that only AT-MSC-CM (*p* = 0.04) and WJ-MSC-CM (*p* = 0.01) induced cell migration in dermal fibroblasts (Figure 6E), whereas BM-MSC-CM did not exert any effect. In gingival fibroblasts, only WJ-MSC-CM stimulated cell migration towards 5% FCS (Figure 6F). Migration assay towards high concentrations of FCS (10% FCS) proved once again that WJ-MSC-CM improved overall performance of cell migration, both in dermal fibroblast (*p* = 0.06) and gingival fibroblasts (*p* = 0.01), as shown in Figures 6G, H, respectively.

All together, these data indicated different effects of tissue-specific MSC-CM on dermal and gingival fibroblast migration probably due to the different content of cytokines and chemokines. Despite the high content of chemotactic factors, BM-MSC-CM induce dermal fibroblast migration only towards low amounts of FCS (1% and 2.5% FCS), without any influence on gingival fibroblasts. It is conceivable that this behaviuor is linked to the high concentration of SDF-1, an important factor for tissue specific recruitment and retention of CXCR4^+^ cells. Indeed, CXCR4 antagonists are used to promote mobilization of hematopoietic stem and progenitor cells from BM niche for transplantation purposes (Rettig et al., 2012). Conversely, WJ-MSC-CM stimulate both dermal and gingival fibroblast migration, towards increasing concentrations of FCS, probably due to high contents of G-CSF and GM-SCF, as previously demonstrated.

### WJ-MSC-CM has tissue repairing properties

Wound healing begins with deposition of a transient ECM composed by fibronectin (FN), vitronectin (VN), and platelets (Chester and Brown, 2017), while collagen (CG) becomes important during re-epithelialization, granulation tissue formation, and neovascularization (Solarte David et al., 2022). Excessive production and deposition of type I collagen leads to tissue fibrosis, as occurs in SSc (van Caam et al., 2018). As MSCs can orchestrate ECM deposition during wound healing, we analyzed the effects of AT-MSC-CM, BM-MSC-CM, and WJ-MSC-CM on VN, FN, and type I collagen deposition in dermal and gingival fibroblasts by ELISA. As a positive control, cells were treated with TGF-β, that induces the expression of ECM proteins (Verrecchia and Mauviel, 2002).

**FIGURE 7:**
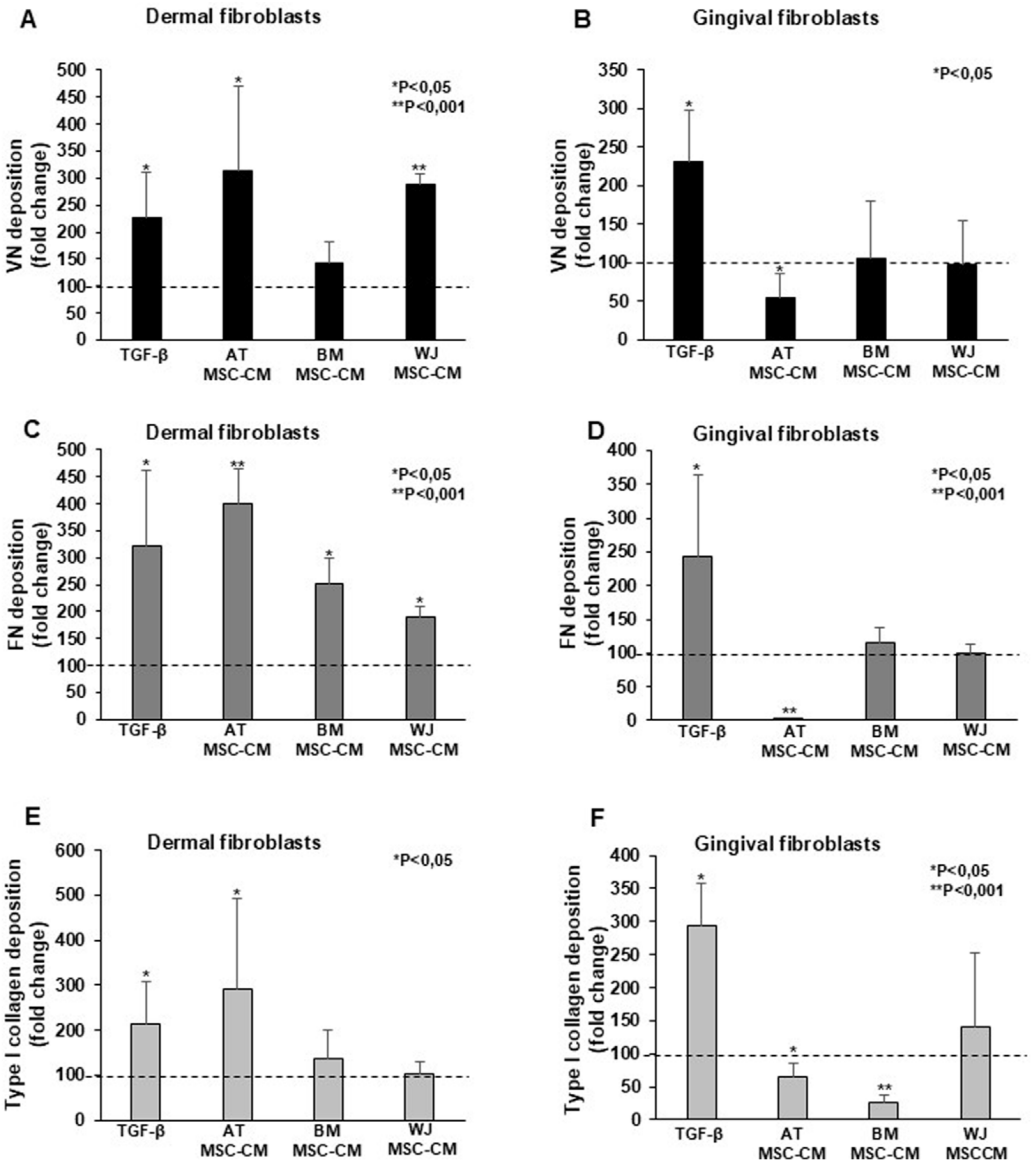
Effects of AT-MSC-CM, BM-MSC-CM, and WJ-MSC-CM on ECM deposition in dermal and gingival fibroblasts. **(A)** Dermal fibroblasts and **(B)** gingival fibroblasts were treated with TGF-β, AT-MSC-CM, BM-MSC-CM, and WJ-MSC-CM for 24 h and analyzed for the cellular VN deposition. The data were expressed as a fold change against untreated cells. Error bars represent standard deviation of the mean of triplicate samples within one experiment. *p < 0.05; **p < 0.001. **(C)** Dermal fibroblasts and **(D)** gingival fibroblasts were treated with TGF-β, AT-MSC-CM, BM-MSC-CM, and WJ-MSC-CM for 24 h and analyzed for the cellular FN deposition. The data were expressed as a fold change against untreated cells. Error bars represent standard deviation of the mean of triplicate samples within one experiment. *p < 0.05; **p < 0.001. **(E)** Dermal fibroblasts and **(F)** gingival fibroblasts were treated with TGF-β, AT-MSC-CM, BM-MSC-CM, and WJ-MSC-CM for 24 h and analyzed for the cellular type I collagen deposition. The data were expressed as a fold change against untreated cells. Error bars represent standard deviation of the mean of triplicate samples within one experiment. *p < 0.05; **p < 0.001.

AT-MSC-CM and WJ-MSC-CM significantly induced VN deposition in dermal fibroblasts (Figure 7A), while not in gingival cells (Figure 7B). AT-MSC-CM, BM-MSC-CM, and WJ-MSC-CM were able to stimulate dermal fibroblasts (Figure 7C), but not gingival fibroblasts (Figure 7D) in which inhibitory effects of AT-MSC-CM on FN induction were found. Effects of AT-MSC-CM, BM-MSC-CM, and WJ-MSC-CM on type I collagen deposition in dermal and gingival fibroblasts are reported in Figures 7E, F respectively. In dermal fibroblasts, only AT-MSC-CM induced type I collagen deposition, whereas in gingival fibroblasts no deposition was found after treatments, as AT-MSC-CM and BM-MSC-CM exerted an inhibitory effect.

AT-MSC-CM, BM-MSC-CM, and WJ-MSC-CM had heterogenous effects on ECM deposition in dermal and gingival fibroblasts. WJ-MSC-CM could be a promising tool for local treatment of SSc-associated skin fibrosis and digital ulcers, as this CM promoted VN and FN deposition, while not pathologic type I collagen, promoted by AT-MSC-CM. Moreover, BM-MSC-CM could be considered as valid alternative to WJ-MSC-CM, although only FN deposition was induced.

### WJ-MSC-CM enhances the ability of lesional SSc fibroblasts to produce pro-angiogenic factors

As we demonstrated that CB-MSC-CM content was poor in cytokines and growth factors, and AT-MSC-CM induced a fibrotic phenotype in dermal fibroblasts, these media were excluded from further studies, while we focused on BM-MSC-CM and WJ-MSC-CM as a potentially therapeutic tool in SSc management. Indeed, there are no approved medications for SSc digital ulcerations and studies are required to identify the optimal local wound bend management strategy (Campochiaro et al., 2023). Therefore, we isolated SSc fibroblasts from skin of 3 SSc patients to analyze the effects of BM-MSC-CM and WJ-MSC-CM on primary cells that offer more biologically relevant models for studying the efficacy of cell-free regenerative therapy.

As a first step, we focused on vasculopathy and loss of angiogenesis as the main pathogenetic features of SSc. In digital ulcers, vasculopathy leads to progressive occlusion of the blood vessels, resulting in reduced capillary density, hypoxia, necrosis and tissue loss (Abraham et al., 2009).

**FIGURE 8:**
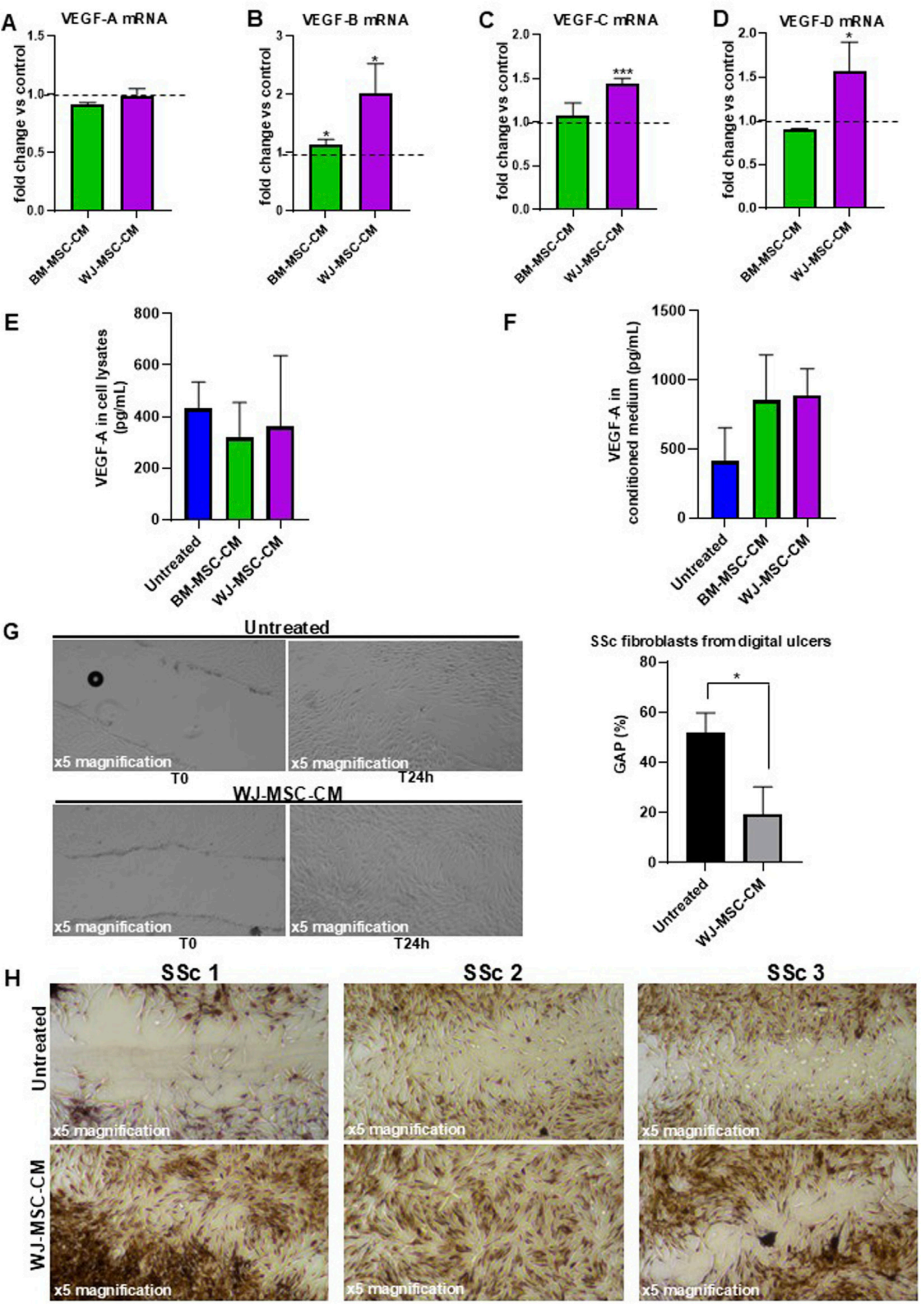
Effects of BM-MSC-CM and WJ-MSC-CM on SSc fibroblasts. **(A)** VEGF-A mRNA expression, **(B)** VEGF-B mRNA expression, **(C)** VEGF-C mRNA expression, **(D)** VEGF-D mRNA expression in SSc fibroblasts treated or not with BM-MSC-CM and WJ-MSC-CM for 24 h at 37°C in a humidified (5% CO_2_) incubator. mRNA expression levels expressed as fold change (%) over control (untreated cells). **(E)** Cellular lysate and **(F)** conditioned medium from SSc fibroblasts, treated or not with BM-MSC-CM and WJ-MSC-CM for 24 h at 37°C in a humidified (5% CO_2_) incubator, were analyzed for protein expression of VEGF-A by ELISA. Values are mean ± SEM of three SSc patients. **(G)** Primary dermal fibroblasts isolated from digital ulcers of SSc patient were treated with basal medium and WJ-MSC-CM for 24 h and analyzed for their capacity to migrate into wounds created by using a sterile pipette tip. Scratch images were acquired using inverted microscope and ×4 magnification. Data were plotted and expressed as a percentage of the length of wound size over T0 (assumed as 100%). Error bars represent standard deviation of the mean of triplicate measurements within one experiment. *p < 0.05; **p < 0.001. **(H)** Masson’s trichrome staining to qualitatively compare wound healing performance and collagen production between control and treated group of primary skin fibroblasts isolated from 3 SSc patients. The microscopic images were observed under an inverted microscope (Leica DMi1) equipped with camera (FLEXACAM C1, Leica) at ×4 magnification.

To investigate whether BM-MSC-CM and WJ-MSC-CM act as a positive modulator of angiogenesis in SSc fibroblasts, we treated the cells with BM-MSC-CM and WJ-MSC-CM for 24 h and measured the mRNA expression levels of several proangiogenic mediators (VEGF-A, VEGF-B, VEGF-C, VEGF-D). As reported in Figures 8A–D, BM-MSC-CM did not exert any effect on mRNA expression of proangiogenic mediators. Consistently, WJ-MSC-CM treatment has no effect on VEGF-A, but it significantly increased mRNA expression of VEGF-B, VEGF-C, and VEGF-D.

To examine whether the VEGF-A transcription levels correlated with VEGF-A protein levels in SSc fibroblasts, we performed an ELISA assay on cell lysates and conditioned media from SSc fibroblasts treated or not with BM-MSC-CM and WJ-MSC-CM, thus also providing a quantitative measurement of VEGF-A. As shown in Figure 8E, no effect on VEGF-A protein expression in cell lysates was detectable after treatment with BM-MSC-CM (318 ± 137 pg/mL) and WJ-MSC-CM (361 ± 275 pg/mL), as compared to untreated cells (431 ± 102 pg/mL). However, increased VEGF-A expression level was found in conditioned media from SSc fibroblasts treated with BM-MSC-CM (852 ± 329 pg/mL) and WJ-MSC-CM (891 ± 189 pg/mL), as compared to untreated cells (409 ± 244 pg/mL) (Figure 8F). Probably, MSC-CM can induce VEGF-A expression in SSc fibroblasts in a time-dependent manner, as demonstrated by VEGF-A accumulation in their cell culture supernatants. It is important to underline that increased VEGF-A expression level in BM-MSC-CM-treated cells could also derive from BM-MSCs which release large quantities of VEGF-A, as previously demonstrated (Figure 3B). Since no VEGF-A could be produced by WJ-MSCs (Figure 3B), VEGF-A increase in cell culture supernatants was effectively due to secretion from SSc fibroblasts after conditioning by WJ-MSC-CM. It was evident from these data that WJ-MSC-CM assure a promising agent in the treatment of SSc skin manifestations.

### WJ-MSC-CM enhances the ability of lesional SSc fibroblasts to migrate into the wounds

To further validate the therapeutic potential of WJ-MSC-CM and propose it as a local wound care option for digital ulcers in SSc, we analyze the effects of WJ-MSC-CM on scratch wound healing in SSc fibroblasts isolated from digital ulcers. As shown in Figure 8G, WJ-MSC-CM-treated cells presented significantly shorter wound length compared to untreated cells (after 24 h treatment/T0, 19.3% vs. 52%-fold change, WJ-MSC-CM treated vs. untreated cells; *p* < 0.05).

Intrigued by this data, we pursued our study by performing Masson’s trichrome staining on scratched SSc fibroblasts pre-treated with WJ-MSC-CM for 24 h to better evaluate wound healing performance, fibroblast morphology, and ECM quality. As shown in Figure 8H, the wounded area is much larger and more evident in untreated fibroblasts as compared to treated cells 24 h after scratching. In the wounded area of untreated cells, poor cellularity and a predominance of collagen (blue staining) were observed. Specifically, untreated cells morphologically appeared irregular, distorted, fusiform, star and web-shaped, and strongly positive for collagen. Conversely, WJ-MSC-CM treatment significantly stimulated cell migration and proliferation of SSc fibroblasts in wounded areas, thus demonstrating that it improves healing rates in wound processes. Notably, diverse fibrous protein including collagen, fibrin, fibronectin, and keratin (red staining) were detected in WJ-MSC-CM-treated cells.

Finally, WJ-MSC-CM could significantly improve wound healing in SSc-derived fibroblasts, thus confirming its potential therapeutic use in digital ulcers management.

## Discussion

Beneficial effects of MSCs have been thought for decades to be related due to their migration to sites of injury, where they differentiated in specialized cells to restore damaged tissue. However, only a small number of cells engraft and survive in damaged tissues, while therapeutic MSCs effects are mainly mediated by secretome components and properties (Vizoso et al., 2017; Chang et al., 2021; Katagiri et al., 2017; Legiawati et al., 2023). Therefore, cell-free based therapy represents a promising therapeutic strategy, as MSC-CM possesses immunomodulatory and antifibrotic properties (Li et al., 2024; Nethi et al., 2023). Based on this evidence, we explored therapeutic potentials in SSc of several sources of MSC-CM, by first analyzing their compositions and functions and then their wound healing effects.

In tissue engineering and clinical applications, the main studied MSC source is the BM, although their harvest requires invasive procedures, and cultured cell number and proliferation/differentiation decrease over time (Mastrolia et al., 2019). In this study, we first isolated and cultured MSCs from AT, BM, WJ, and CB, and then MSC-CM were collected to perform a comparative analysis of cytokine composition. BM-MSC-CM and WJ-MSC-CM were major sources of cytokines and growth factors, especially proangiogenic agents including PDGF, HGF, FGF, MCP-1, IL-8, and IL-6, and these media could represent an ideal device for clinical applications in SSc. Conversely, CB-MSC-CM was a poor source of cytokines and growth factors, while AT-MSC-CM had high IL-6 levels. Moreover, VEGF, the key angiogenic molecule, was exclusively contained in BM-MSC-CM. Therefore, based on their cytokine and growth proangiogenic factor composition, BM-MSC-CM and WJ-MSC-CM might be employed to ameliorate vascularization of avascular skin areas (also known as desertification), to modulate altered immune responses through IL-10 and IL-2 secretion, and to halt fibrosis in SSc (Margiana et al., 2022; Yu et al., 2022). BM-MSC-CM and WJ-MSC-CM were also characterized by high levels of HGF, that is strongly associated with antifibrotic effects, although WJ-MSC-CM showed concomitant high TGF-β1 levels, a pro-fibrotic agent. However, TGF-β1 could also regulate cell homeostasis, resolution of inflammation, and injury repair (Ramirez et al., 2014). Therefore, further investigations are needed to elucidate the exact effects of WJ-MSC-derived TGF-β1 and its interaction with other released factors.

BM-MSC-CM was characterized by a broader spectrum of chemokines, compared to AT-MSC-CM, WJ-MSC-CM, and CB-MSC-CM. Notably, SDF-1 was exclusively contained in BM-MSC-CM, as described for VEGF. After binding to its receptor (CXCR4), SDF-1 can act as an attractant for stem cells during tissue repair, but can also act as a pro-inflammatory molecule, contributing to injury (Bragg et al., 2019). Therefore, medium composition appeared to be the principal variable to influence tissue regeneration, as BM-MSC-CM exclusively included two important factors, that mediate stem cell migration to the site of injury (SDF-1) on one hand and promote tissue repair through neoangiogenesis and growth factor release (VEGF) on the other hand. Of note, chemoattraction of CXCR4^+^ cells (mainly BM-derived stem cells) could further enhance SDF-1 and VEGF release at the site of injury. Moreover, cell proliferation and migration are essential to proper wound healing, and TGF-β1 might support these events, as described in 3D high-density cultures of BM- and WJ-MSCs, especially in this latter cell type (Lamparelli et al., 2022).

Overall, single secreted factors and their interactions can explain the functional relationships between MSCs and the tissue of origin. The different expression of trophic factors secreted by AT-MSCs, BM-MSCs, WJ-MSCs, and CB-MSCs revealed the presence of a “tissue-specific cytokine signature” that distinguishes the MSCs of each compartment and most likely influences their biological activity. Adipose stroma could be influenced by metabolic and hormonal status, but in absence of pathogenetic stimuli AT-MSCs appear in a relatively immune-inert status, as shown by low secretion levels in AT-MSCs isolated from mammary tissue of healthy woman. Bone marrow stroma consists of a heterogenous population of cells, including multipotent MSCs, endothelial cells, adipocytes, osteocytes, and osteoblasts. In addition, bone marrow stroma supports hematopoiesis and regulates tissue regeneration (Nombela-Arrieta and Isringhausen, 2017). Therefore, the secretion profile and biological features of BM-MSCs could be influenced by a dynamic environment that significantly stimulates the release of cytokines, growth factors and chemokines. The observation that WJ-MSCs produce larger amounts of cytokines and growth factors could be explained by the composition and function of jelly-like substance inside the umbilical cord. Jelly-like substance is mucous tissue that protects the umbilical arteries and vein and is a reservoir of activated growth factors, matrix components or precursor forms, that provide a strong mechanical resistance, elasticity and a high degree of hydration to prevent the umbilical cord vessels from occlusion (Gupta et al., 2020). It is conceivable that this environment strongly influences the biology of WJ-MSCs that show immunoregulatory capacities and high regenerative potential by an intense paracrine action, as demonstrated by our results. Surprisingly, WJ-MSCs exhibit different morphological features and secretion profile as compared to CB-MSCs, despite the same perinatal origin. Further studies are required to understand the biological significance of the different characteristics between WJ-MSCs and CB-MSCs, and to determine whether WJ-MSCs possess better properties for tissue regeneration.

According to published literature, we showed that WJ-MSC-CM could be an excellent source for regenerative medical approaches, as both BM-MSC-CM and WJ-MSC-CM induced fibroblast growth, proliferation, cell cycle progression, and protection from cellular senescence, but only WJ-MSC-CM significantly enhanced dermal and gingival fibroblast migration. In addition, BM-MSC-CM only induced FN in dermal fibroblasts, conversely WJ-MSC-CM contributed to wound repair by promoting provisional matrix proteins deposition, including VN and FN, while not pathologic type I collagen, and wound closure/re-epithelialization and probably angiogenesis and scar tissue formation. These effects are very promising for clinical application of WJ-MSC-CM in SSc therapies, as the hallmark of SSc skin fibrosis is represented by excess deposition and accumulation of type I collagen. Therefore, a regenerative approach that can enhance and restore ECM content without affecting collagen deposition is attractive for therapies of collagenopathies, especially SSc, and WJ-MSC-CM are the best source for clinical use in SSc.

Finally, WJ-MSC are easier to isolate from the gelatinous layer of umbilical cord without an invasive and painful procedure, exhibit higher proliferation potential than other cells isolated from other tissues, and no ethical issues are present (Kim et al., 2013). Moreover, WJ-MSCs should be the preferred cell source for their stemness properties and degree of differentiation potential (Subramanian et al., 2015). In addition, several preclinical studies selected WJ-MSCs as an excellent source for treatment of fibrosis-associated diseases, including pulmonary fibrosis (Saleh et al., 2022).

All this evidence opens a new perspective in SSc treatment using WJ-MSC-CM; however, several limitations are still unquestioned, including cell culture procedure standardization, appropriate dose, administration route, and frequency of treatment. Moreover, more trials are needed to assess their efficacy in clinical settings.

In conclusion, WJ-MSC-CM contains a wide range of bioactive factors, with potent wound healing and angiogenesis functions. Therefore, WJ-MSC-CM could represent a novel potential cell-free therapeutic option, requiring minimal manipulation procedures and feasible industrial approach. Therefore, future studies will investigate a biocompatible matrix that allows controlled release of WJ-MSC-CM thereby providing a new therapeutic option for local treatment of SSc skin ulcers.

## Data Availability

The raw data supporting the conclusions of this article will be made available by the authors, without undue reservation.
