# Supplementary material for: Cell-free regenerative medicine: identifying the best source of mesenchymal stem cells for skin therapy in Systemic Sclerosis
Source: Front Cell Dev Biol. 2025 Feb 19;13:1518412. doi: 10.3389/fcell.2025.1518412 (PMC11880212; doi:10.3389/fcell.2025.1518412)
Supplement: Supplementary file 1 [file DataSheet1.docx]

Supplementary Material


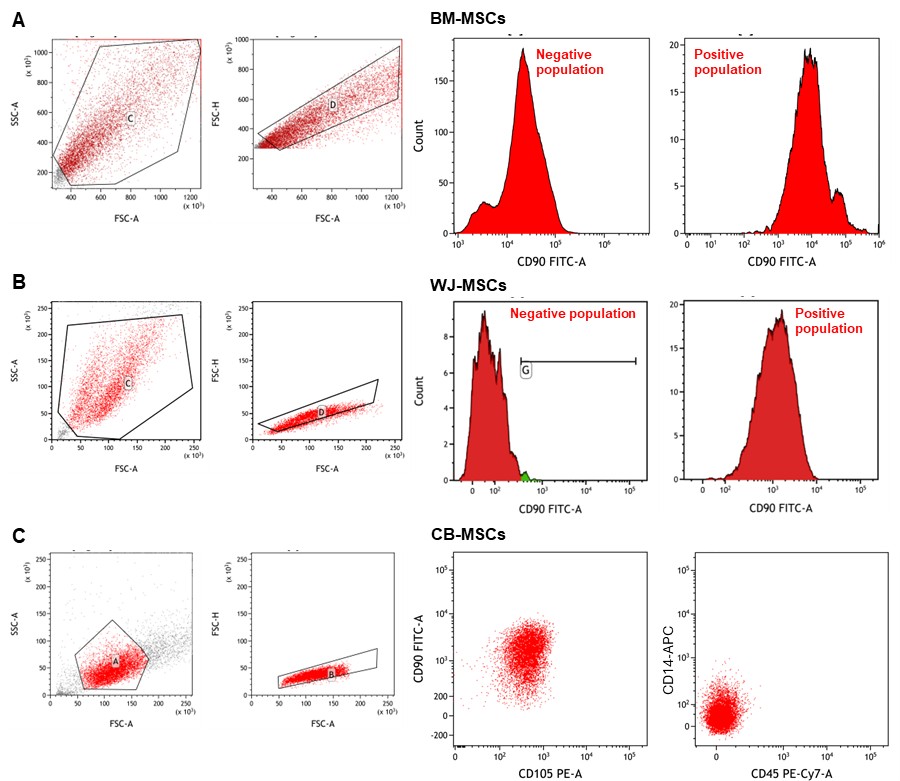


**Supplementary Figure 1**. Flow cytometry gating strategy. After post-acquisition compensation using FlowJo software (v.10.7.1, LLC, BD Biosciences, Franklin Lakes, NJ, USA), AT-MSCs (A), BM-MSCs (B), and WJ-MSCs (C) were first identified using linear parameters (forward scatter area [FSC-A] vs side scatter area [SSC-A], and double cells were excluded (FSC-A vs FSC-W) and double cells were excluded (FSC-A vs. FSC-H). Expression of each marker on single cells was reported using histograms and an unstained sample as negative control.
